# Polymorphism of *NOS3* gene and its association with essential hypertension in Guizhou populations of China

**DOI:** 10.1371/journal.pone.0278680

**Published:** 2023-02-09

**Authors:** Ruichao Li, Ansu Zhao, Xiaoyan Diao, Juhui Song, Chanjuan Wang, Yanhong Li, Xiaolan Qi, Zhizhong Guan, Ting Zhang, Yan He

**Affiliations:** 1 Key Laboratory of Endemic and Ethnic Diseases, Ministry of Education & Key Laboratory of Medical Molecular Biology of Guizhou Province, & Collaborative Innovation Center for Prevention and Control of Endemic and Ethnic Regional Diseases Co-constructed by the Province and Ministry, Guizhou Medical University, Guiyang, Guizhou, China; 2 Department of Cardiovascular Medicine, Affiliated Hospital of Guizhou Medical University, Guiyang, China; Keio University School of Medicine, JAPAN

## Abstract

**Objective:**

A case-control study was conducted to evaluate the relationship between endothelial nitric oxide synthase (*NOS3*) gene polymorphism and essential hypertension in the Han, Miao, and Buyi populations in Guizhou China.

**Methods:**

DNA was collected from the blood samples of 353 essential hypertension patients and 342 healthy controls from Guizhou province of China. Eight polymorphisms of the *NOS3* gene were genotyped using the Sequenom MassARRAY platform. For genetic analysis, SPSS 26.0, Haploview, SNPStats, SHEsis, and MDR were utilized.

**Results:**

All SNPs (rs11771443, rs1808593, rs753482, rs3918186, rs3918188, rs3918227, rs7830, and rs891512) satisfied the Hardy-Weinberg equilibrium test (*P* > 0.05). The allele and genotype frequencies of rs7830 and rs1808593 in case-control groups demonstrated significant differences (*P* < 0.05). Compared to the TT genotype of rs1808593, the TG or GG genotype reduced the risk of hypertension in the Miao population (OR = 0.410, 95% CI: 0.218–0.770, *P* = 0.006). Compared to the GG or GT genotype of rs7830, the TT genotype increased the risk of hypertension in the overall populations (OR = 1.716, 95%CI: 1.139–2.586, *P* = 0.010). The CATT (rs3918227-rs391818186-rs1808593-rs7830) haplotype was a risk factor for hypertension in the Miao and Han populations (OR = 1.471, 95%CI: 1.010–2.143, *P* = 0.044 and OR = 1.692, 95%CI: 1.124–2.545, *P* = 0.011). The CAGG haplotype in the Miao population was a protective factor against hypertension (OR = 0.555, 95%CI: 0.330–0.934, *P* = 0.025). The rs3918188, rs1808593, and rs7830 in the Miao population showed an interaction effect on hypertension (*P* < 0.001). The rs11771443, rs3918188, and rs7830 in the Buyi and Han populations showed an interaction effect on hypertension (*P* = 0.013 and *P* < 0.001).

**Conclusion:**

The single nucleotide polymorphisms rs1808593 and rs7830 of *NOS3* gene are associated with essential hypertension in Guizhou ethnic populations.

## Introduction

Essential hypertension (EH) is defined by increased blood pressure of unexplained origin and accounts for approximately 95% of all hypertension cases. The incidence rate of hypertension in the world population has increased from 26.4% in 2000 to 31.1% in 2010 [1]. Besides, the incidence rate of hypertension in the adult population of China was 23.2% (approximately 244.5 million) according to the results of the Chinese Hypertension Survey from 2012 to 2015, especially in Guizhou province [2]. Hypertension and cardiovascular illness have become a major public health concern in recent years because of the social pressure, environmental pressure, and the aging population in China [3]. Thus, hypertension has great research significance.

EH is a complex multifactorial disease influenced by genetic variables, accounting for 30 to 50 percent of changes in blood pressure [4]. A single nucleotide variation at a specific place in a genomic DNA sequence is referred to as a single nucleotide polymorphism (SNP). The rate of effective SNPs related to blood pressure is reportedly high in genome-wide association studies (GWAS). Blood pressure regulation is heavily influenced by genetics [5]. The endothelial nitric oxide synthase (*eNOS*) gene, also known as nitric oxide synthase 3 (*NOS3*), is found on human chromosome 7 in the 7q36.1 region, and comprises 23,572 base pairs and 28 exons [6, 7]. The *eNOS* gene encodes NOS3, which catalyzes the conversion of l-arginine to endothelial nitric oxide, a potent vasodilator that helps maintain cardiovascular homeostasis by dilating blood vessels and lowering baseline blood pressure [8]. Regulation at the transcriptional, post-transcriptional, and post-translational levels influence *NOS3* expression and activity. SNP has been shown to influence the production of eNO [9]. However, no consensus has been reached on the relationship between *NOS3* gene polymorphism and hypertension is debatable. Shoji M. et al. [10] found that rs1799983 is significantly linked to hypertension in the Japanese population. Moreover, the rs1799983 polymorphism has been linked to hypertension in the Asian population [11]. However, it has been reported that rs1799983 is not linked to EH in the Han population in Northern China [12]. Few studies have hitherto investigated the relationship between *NOS3* gene polymorphism and EH in the Guizhou population.

It is well-established that harnessing the molecular weight for genetic identification in SNP typing can be conducted by MassARRAY flight mass spectrometry, a high-throughput, accurate, and sensitive method in genetic testing [13]. Therefore, it is of great value to use flight mass spectrometry to study the correlation between *NOS3* polymorphism and EH in the Guizhou populations to provide a theoretical foundation for future pathogenesis research as well as prevention and control strategies for EH.

## Methods

### Study participants

From 2016 to 2018, 353 cases of EH were screened from the outpatient, inpatient, and physical examination populations at the Department of Cardiology of Guizhou Medical University’s Affiliated Hospital and community hospitals in Leishan and Libo County, including 122 Han, 111 Miao, and 120 Buyi participants. At the same time, 342 healthy subjects that underwent routine medical checkups at the Affiliated Hospital of Guizhou Medical University and Leishan/Libo County Community Hospital, including 110 Han, 112 Miao and 120 Buyi, were selected as the control group. PASS15 (NCSS, LLC. Kaysville, Utah, USA) software showed that the statistical power of this study was over 80%. We recorded the gender, age, ethnicity, blood pressure, height, and weight of all participants. The subjects were classified as low BMI (BMI < 18.5kg/m^2^), normal BMI (18.5kg/m^2^ ≤ BMI < 24.0kg/m^2^), overweight (24.0kg/m^2^ ≤ BMI < 28.0kg/m^2^), and obese (BMI ≥ 28.0kg/m^2^) based on the Chinese Adult Overweight and Obesity Prevention and Control Guidelines and the Body Mass Index (BMI) calculation formula (BMI = kg/m^2^). This study was approved by the Ethics Committee of the Affiliated Hospital of Guizhou Medical University (Approval number: [2014] Ethics Review No. 45). All subjects volunteered to participate in the experiment and provided written informed consent.

### Screening standard

The inclusion criteria for the EH group included: (1) Patients over 18 years of age; (2) Blood pressure measured 3 times on different days, resting systolic blood pressure ≥ 140 mmHg (1 mmHg = 0.133 kPa) and/or diastolic blood pressure ≥ 90 mmHg; (3) Patients with normal blood pressure who had been diagnosed with EH and were currently taking antihypertensive drugs, based on the 2010 Chinese guidelines for hypertension prevention and treatment. The study subjects were from Guizhou’s Han, Miao, and Buyi ethnic populations. There was no history of consanguineous marriage within three generations. Patients were excluded for the following reasons: secondary hypertension, congenital heart disease, cardiomyopathy, valvular disease, liver failure, kidney failure, pregnant and lactating women, drug abusers or patients with a history of mental illness.

The inclusion criteria for the control group consisted of (1) Systolic blood pressure < 140 mmHg and diastolic blood pressure < 90 mmHg; (2) No history of hypertension and not taking antihypertensive drugs. All study subjects were from Guizhou’s Han, Miao, and Buyi ethnic populations. There was no history of consanguineous marriage within three generations. The exclusion criteria were the same as the disease groups.

### DNA extraction, quantification, and quality testing

Blood cell DNA was extracted from peripheral venous blood of all subjects, using a human peripheral blood DNA extraction kit (QIAGEN, Germany). The DNA concentration and purity were determined by NanoDrop2000 (Thermo Fisher Scientific Inc.). Then DNA was quantified by 30ng/μl to 96-well plates, with 2μl DNA for electrophoresis on 1.25% agarose gel (170V, 20min). Finally, the gel electrophoresis imager (Major Science) was used to observe the electrophoretic bands for the experimental requirements. SNP detection requirements were: DNA concentration >20 mg/L, A_260_/A_280_ between 1.6 and 2.2. The DNA electrophoresis band was intact without degradation. DNA samples that did not meet the requirements were discarded.

### Primer design and synthesis

To assess the potential risks in gene typing, the homology of SNPs was verified using the UCSC database (http://genome.ucsc.edu/). Finally, eight SNPs of *NOS3* gene were identified: rs11771443, rs3918227, rs3918186, rs3918188, rs753482, rs891512, rs1808593, and rs7830. The Assay Design 4.0 (Agena Bioscience, Inc) program was utilized to evaluate primer design for multiple SNPs. The design parameters were adjusted to meet the optimization criteria based on different sites. Three primers corresponding to each SNP were produced using the PAGE primers purification process, comprising two PCR primers and one single base extension primer, as shown in S1 Table.

### SNP genotyping test

MassARRAY detection platform (Agena Bioscience, Inc.) was used for PCR amplification, shrimp alkaline phosphatase reaction, single base extension reaction, resin purification, chip sampling, and mass spectrometry. The original data and genotypic scatter plot were collected using Typer4.0 (Agena Bioscience, Inc).

### Statistical analysis

The data was analyzed using SPSS26.0 (IBM Corp., Armonk, NY, USA) software. The Student’s t-test was used to compare the continuous data, which were represented as mean ± standard deviation. Qualitative data were expressed as rates, and comparisons between groups were performed using the Chi-Square or Fisher’s exact test. The genetic model’s odds ratio (OR) and 95% confidence interval (95% CI) were obtained using binary logistic regression after adjusting for age, sex, and BMI, which were used to estimate the risk of hypertension. The allele, genotype frequency, and Hardy-Weinberg equilibrium test were calculated using the SNPStats online software (https://snpstats.net/start.htm). Linkage disequilibrium (LD) was analyzed using the Haploview software. The SHEsis online software (http://analysis.bio-x.cn/) was used to construct haplotypes and calculate the OR and 95% CI. MDR3.0.2 software was used to perform SNP-SNP interaction analysis. *P* values for multiple testing were corrected for False discovery rate (FDR) using R software (version 3.6.2, R Foundation for Statistical Computing, Vienna, Austria). A two-tailed test with a *P* < 0.05 revealed a statistically significant difference.

## Results

### Demographic and clinical characteristics of subjects

There was no statistically significant difference in gender or BMI between the hypertension group and the control group (*P* > 0.05). The hypertension group’s mean age was significantly higher than the control group’s (*P* < 0.05), as shown in Table 1. There were statistically significant differences in fasting blood glucose, total cholesterol, and triglyceride between the EH group and control group in the Guizhou Han population (all *P*_*s*_ < 0.05), as shown in S2 Table.

**Table 1:** Basic characteristics of research objects of Miao, Buyi and Han populations in Guizhou.

	Total populations		*P*	Miao population		*P*	Buyi population		*P*	Han population		*P*
	EH (n = 343)	CO (n = 335)		EH (n = 110)	CO (n = 111)		EH (n = 119)	CO (n = 117)		EH (n = 114)	CO (n = 107)	
Gender (Male/female)	164/179	151/184	0.475	58/52	44/67	0.059	61/58	52/65	0.301	45/69	55/52	0.080
Age (years)	58.87±12.85	51.93±13.91	**<0.001**	57.91±13.69	51.15±15.92	**0.001**	60.93±12.91	51.63±13.99	**<0.001**	57.63±11.74	53.07±11.40	**0.004**
Low BMI [n (%)] ^a^	14(4)	17(5)	0.190 ^b^	3(3)	5(4)	0.606	8(7)	8(7)	0.736	3(3)	4(4)	0.507
Normal BMI [n (%)]	156(45)	176(53)		48(44)	55(50)		58(49)	65(56)		50(44)	56(52)	
Overweight [n (%)]	123(36)	105(31)		42(38)	39(35)		41(35)	33(28)		40(35)	33(31)	
Obesity [n (%)]	50(15)	37(11)		17(15)	12(11)		12(10)	11(9)		21(18)	14(13)	

Abbreviations: EH, essential hypertension; CO, control; BMI, Body Mass Index. Measurement data were expressed as mean±SD, and Student’s t-test was used for comparison between groups. Counting data were expressed as frequency (rate), and Chi-Square test or Fisher’s exact test was used for comparison between groups.

^a^, BMI = kg/m^2^, Low BMI: BMI<18.5. Normal BMI: 18.5≤BMI<24.0, Overweight: 24.0≤BMI<28.0, Obesity: BMI≥28.0;

^b^: The *P* values of the Chi-Square test of "2×C contingency table "were adjusted by Bonferroni test.

### Risk factors of essential hypertension in the Guizhou populations

Taking hypertension as the dependent variable and age, gender and BMI as independent variables, a binary multivariate logistic regression model was constructed. For the general population of Guizhou, age, overweight and obesity were independent risk factors for EH (OR = 1.044, 95%CI: 1.031–1.057, *P* < 0.001; OR = 1.611, 95%CI: 1.127–2.302, *P* = 0.009 and OR = 2.065, 95%CI: 1.248–3.418, *P* = 0.005), as shown in Table 2.

**Table 2:** Essential hypertension risk factors in Guizhou populations (n = 678).

Variables	B	SE	Wald	Adjusted OR (95% CI) ^a^	*P*
Age (years)	0.043	0.006	47.049	1.044 (1.031~1.057)	**<0.001**
Female				1	
Male	0.040	0.161	0.063	1.041 (0.759~1.429)	0.802
Normal BMI				1	
Low BMI	-0.188	0.397	0.224	0.829 (0.381~1.804)	0.636
Overweight	0.477	0.182	6.859	1.611 (1.127~2.302)	**0.009**
Obesity	0.725	0.257	7.959	2.065 (1.248~3.418)	**0.005**

Abbreviations: OR, odds ratio; 95%CI, 95% confidence interval.

^a^: Adjusted for age, sex, BMI.

### *NOS3* genotyping of different populations in Guizhou

The Hardy-Weinberg equilibrium test was used to validate all SNPs. The Hardy-Weinberg equilibrium was found in 8 loci (*P* > 0.05), as shown in Table 3. The allele and genotype frequencies of rs1808593 and rs7830 significantly differed between hypertension and control groups (*P* < 0.05). After stratification according to the ethnic group, the allele and genotype frequencies of rs1808593 and rs7830 showed statistical differences between the hypertension group and control group in Guizhou Miao and Han populations, respectively (*P* < 0.05). However, no significant differences were found after adjusting for FDR (*P*>0.05), as shown in S3 Table. There were no loci with significant differences in the Guizhou Buyi population (*P* > 0.05), as shown in Table 3.

**Table 3:** Distribution of *NOS3* alleles and genotypes.

SNPs	Alleles Genotypes	Total populations (EH = 343, CO = 335)		*P* ^a^	*P* ^b^	Miao population (EH = 110, CO = 111)		*P* ^a^	*P* ^b^	Buyi population (EH = 119, CO = 117)		*P* ^a^	*P* ^b^	Han population (EH = 114, CO = 107)		*P* ^a^	*P* ^b^
		CO	EH			CO	EH			CO	EH			CO	EH		
rs11771443	C	413(62%)	430(63%)	0.695	0.856	153(69%)	143(65%)	0.419	0.715	140(60%)	146(61%)	0.778	0.896	120(56%)	141(62%)	0.246	0.310
	T	257(38%)	256(37%)			69(31%)	77(35%)			94(40%)	92(39%)			94(44%)	87(38%)		
	CC	129(39%)	134(39%)			54(49%)	48(44%)			43(37%)	47(39%)			32(30%)	39(34%)		
	CT	155(46%)	162(47%)			45(41%)	47(43%)			54(46%)	52(44%)			56(52%)	63(55%)		
	TT	51(15%)	47(14%)			12(11%)	15(14%)			20(17%)	20(17%)			19(18%)	12(11%)		
	*P*_*hwe*_	0.730	0.910			0.660	0.530			0.700	0.440			0.560	0.110		
rs3918227	C	628(94%)	652(95%)	0.345	0.218	213(96%)	213(97%)	0.800	0.796	212(91%)	220(92%)	0.511	0.291	203(95%)	219(96%)	0.649	0.537
	A	42(6%)	34(5%)			9(4%)	7(3%)			22(9%)	18(8%)			11(5%)	9(4%)		
	CC	293(87%)	310(90%)			102(92%)	103(94%)			95(81%)	102(86%)			96(90%)	105(92%)		
	CA	42(13%)	32(9%)			9(8%)	7(6%)			22(19%)	16(13%)			11(10%)	9(8%)		
	AA	0(0%)	1(1%)			0(0%)	0(0%)			0(0%)	1(1%)			0(0%)	0(0%)		
	*P*_*hwe*_	0.630	0.580			1.000	1.000			0.600	0.500			1.000	1.000		
rs3918186	A	616(92%)	629(92%)	0.921	0.253	205(92%)	205(93%)	0.855	0.556	212(91%)	215(90%)	0.923	0.166	199(93%)	209(92%)	0.722	0.781
	T	54(8%)	57(8%)			17(8%)	15(7%)			22(9%)	23(10%)			15(7%)	19(8%)		
	AA	282(84%)	291(85%)			94(85%)	96(87%)			95(81%)	99(83%)			93(87%)	96(84%)		
	AT	52(16%)	47(14%)			17(15%)	13(12%)			22(19%)	17(14%)			13(12%)	17(15%)		
	TT	1(0%)	5(1%)			0(0%)	1(1%)			0(0%)	3(3%)			1(1%)	1(1%)		
	*P*_*hwe*_	0.710	0.070			1.000	0.400			0.600	0.074			0.410	0.560		
rs3918188	C	430(64%)	451(66%)	0.569	0.696	139(63%)	151(69%)	0.194	0.415	155(66%)	147(62%)	0.338	0.553	136(64%)	153(67%)	0.484	0.511
	A	240(36%)	235(34%)			83(37%)	69(31%)			79(34%)	91(38%)			78(36%)	75(33%)		
	CC	139(41%)	153(45%)			46(41%)	53(48%)			52(44%)	48(40%)			41(38%)	52(46%)		
	CA	152(45%)	145(42%)			47(42%)	45(41%)			51(44%)	51(43%)			54(50%)	49(43%)		
	AA	44(13%)	45(13%)			18(16%)	12(11%)			14(12%)	20(17%)			12(11%)	13(11%)		
	*P*_*hwe*_	0.810	0.280			0.310	0.660			0.840	0.330			0.410	0.830		
rs753482	A	660(99%)	676(99%)	0.958	0.957	219(99%)	218(99%)	1.000	1.000	231(99%)	233(98%)	0.724	0.722	210(98%)	225(99%)	0.717	0.715
	C	10(1%)	10(1%)			3(1%)	2(1%)			3(1%)	5(2%)			4(2%)	3(1%)		
	AA	325(97%)	333(97%)			108(97%)	108(98%)			114(97%)	114(96%)			103(96%)	111(97%)		
	AC	10(3%)	10(3%)			3(3%)	2(2%)			3(3%)	5(4%)			4(4%)	3(3%)		
	CC	0(0%)	0(0%)			0(0%)	0(0%)			0(0%)	0(0%)			0(0%)	0(0%)		
	*P*_*hwe*_	1.000	1.000			1.000	1.000			1.000	1.000			1.000	1.000		
rs891512	G	637(95%)	661(96%)	0.283	0.366	216(97%)	218(99%)	0.285	0.280	217(93%)	221(93%)	0.959	1.000	204(95%)	222(97%)	0.312	0.495
	A	33(5%)	25(4%)			6(3%)	2(1%)			17(7%)	17(7%)			10(5%)	6(3%)		
	GG	303(90%)	318(93%)			105(95%)	108(98%)			100(85%)	102(86%)			98(92%)	108(95%)		
	GA	31(9%)	25(7%)			6(5%)	2(2%)			17(15%)	17(14%)			8(7%)	6(5%)		
	AA	1(0%)	0(0%)			0(0%)	0(0%)			0(0%)	0(0%)			1(1%)	0(0%)		
	*P*_*hwe*_	0.560	1.000			1.000	1.000			1.000	1.000			0.200	1.000		
rs1808593	T	523(78%)	572(83%)	**0.013**	**0.023**	175(79%)	193(88%)	**0.015**	**0.022**	190(81%)	196(82%)	0.812	0.230	158(74%)	183(80%)	0.114	0.228
	G	147(22%)	114(17%)			47(21%)	27(12%)			44(19%)	42(18%)			56(26%)	45(20%)		
	TT	200(60%)	239(70%)			68(61%)	86(78%)			74(63%)	81(68%)			58(54%)	72(63%)		
	TG	123(37%)	94(27%)			39(35%)	21(19%)			42(36%)	34(29%)			42(39%)	39(34%)		
	GG	12(4%)	10(3%)			4(4%)	3(3%)			1(1%)	4(3%)			7(7%)	3(3%)		
	*P*_*hwe*_	0.260	0.850			0.780	0.200			0.072	0.760			1.000	0.560		
rs7830	G	406(61%)	371(54%)	**0.016**	**0.035**	114(51%)	94(43%)	0.071	0.192	149(64%)	150(63%)	0.924	0.950	143(67%)	127(56%)	**0.019**	**0.038**
	T	264(39%)	315(46%)			108(49%)	126(57%)			85(36%)	88(37%)			71(33%)	101(44%)		
	GG	122(36%)	106(31%)			29(26%)	20(18%)			46(39%)	47(39%)			47(44%)	39(34%)		
	GT	162(48%)	159(46%)			56(50%)	54(49%)			57(49%)	56(47%)			49(46%)	49(43%)		
	TT	51(15%)	78(23%)			26(23%)	36(33%)			14(12%)	16(13%)			11(10%)	26(23%)		
	*P*_*hwe*_	0.910	0.230			1.000	1.000			0.690	1.000			0.830	0.180		

Abbreviations: EH, essential hypertension; CO, control; hwe: Hardy-Weinberg equilibrium test. Chi-Square test or Fisher’s exact test was used for counting data comparison.

^a^: *P* value of allele;

^b^: *P* value of genotype.

### Genetic pattern analysis of *NOS3*

Loci with statistically significant differences in the allele frequency between hypertension and control groups (*P* < 0.05) underwent genetic pattern analysis.

In the overall Guizhou populations, the dominant, overdominant, and log-additive genetic patterns of rs1808593 were statistically significant (*P* < 0.05). Multivariate logistic regression analysis adjusted for age, gender, and BMI showed that the TG or GG genotype was associated with reduced risk of hypertension compared to TT genotype (OR = 0.635, 95% CI: 0.454–0.887, *P* = 0.008); the TG genotype with reduced risk compared to TT or GG genotype (OR = 0.654, 95% CI: 0.465–0.919, *P* = 0.014); and allele G with reduced risk compared to allele T (OR = 0.682, 95% CI: 0.509–0.914, *P* = 0.010). Similarly, significant recessive and log-additive genetic patterns were observed for SNP rs7830 (*P* < 0.05). After adjusting by age, gender, and BMI during multivariate logistic regression analysis, the TT genotype was associated with an increased risk of hypertension compared to GG or GT genotype (OR = 1.716, 95% CI: 1.139–2.586, *P* = 0.010). Moreover, allele T increased the risk of hypertension compared to allele G (OR = 1.323, 95% CI: 1.056–1.657, *P* = 0.015).

In the Guizhou Miao population, the dominant, overdominant, and log-additive genetic patterns of rs1808593 were statistically significant (*P* < 0.05). Multivariate logistic regression analysis adjusted for age, gender, and BMI showed that the TG or GG genotype was associated with a reduced risk of hypertension compared to the TT genotype (OR = 0.410, 95% CI: 0.218–0.770, *P* = 0.006) and allele G with reduced risk compared to allele T (OR = 0.496, 95% CI: 0.284–0.864, *P* = 0.013). The recessive pattern of rs7830 was statistically significant in the Guizhou Han population (*P* < 0.05). Increased risk of hypertension was found in the TT genotype compared to GG or GT genotype (OR = 2.579, 95% CI: 1.203–5.525, *P* = 0.015). However, when age, sex, BMI, fasting blood glucose, cholesterol, and triglyceride were included, no significant differences were observed (*P* > 0.05), as shown in Table 4.

**Table 4:** Genetic patterns analysis of *NOS3* gene in Guizhou populations.

SNPs	Nation	Genetic patterns	Genotype	Control	EH	B	SE	Wald	Adjusted OR (95% CI) ^a^	*P*
rs1808593	Total populations	Dominant	TT	200(60%)	239(70%)				1	
			TG-GG	135(40%)	104(30%)	-0.455	0.170	7.109	0.635 (0.454–0.887)	**0.008**
		Overdominant	TT-GG	212(63%)	249(73%)				1	
			TG	123(37%)	94(27%)	-0.425	0.174	5.985	0.654 (0.465–0.919)	**0.014**
		Log-additive	---	---	---	-0.383	0.149	6.572	0.682 (0.509–0.914)	**0.010**
	Miao population	Dominant	TT	68(61%)	86(78%)				1	
			TG-GG	43(39%)	24(22%)	-0.892	0.322	7.677	0.410 (0.218–0.770)	**0.006**
		Overdominant	TT-GG	72(65%)	89(81%)				1	
			TG	39(35%)	21(19%)	-0.917	0.333	7.592	0.400 (0.208–0.767)	**0.006**
		Log-additive	---	---	---	-0.702	0.283	6.134	0.496 (0.284–0.864)	**0.013**
rs7830	Total populations	Recessive	GG-GT	284(85%)	265(77%)				1	
			TT	51(15%)	78(23%)	0.54	0.209	6.677	1.716 (1.139–2.586)	**0.010**
		Log-additive	---	---	---	0.28	0.115	5.945	1.323 (1.056–1.657)	**0.015**
	Han population	Recessive	GG-GT	96(90%)	88(77%)				1	
			TT	11(10%)	26(23%)	0.454	0.441	1.062	1.574 (0.664–3.733)	0.303 ^b^

Gene X (Major allele: A, Minor allele: B); Codominant (AA vs AB or AA vs BB); Dominant (AA vs AB+BB); Recessive (AA+AB vs BB); Overdominant (AA +BB vs AB); Log-additive (increasing ordinal variable analysis of the B allele).

^a^, adjust age, sex, BMI;

^b^, adjust age, sex, BMI, fasting blood-glucose, cholesterol, triglyceride.

### Linkage disequilibrium and haplotype analysis

**Fig 1:**
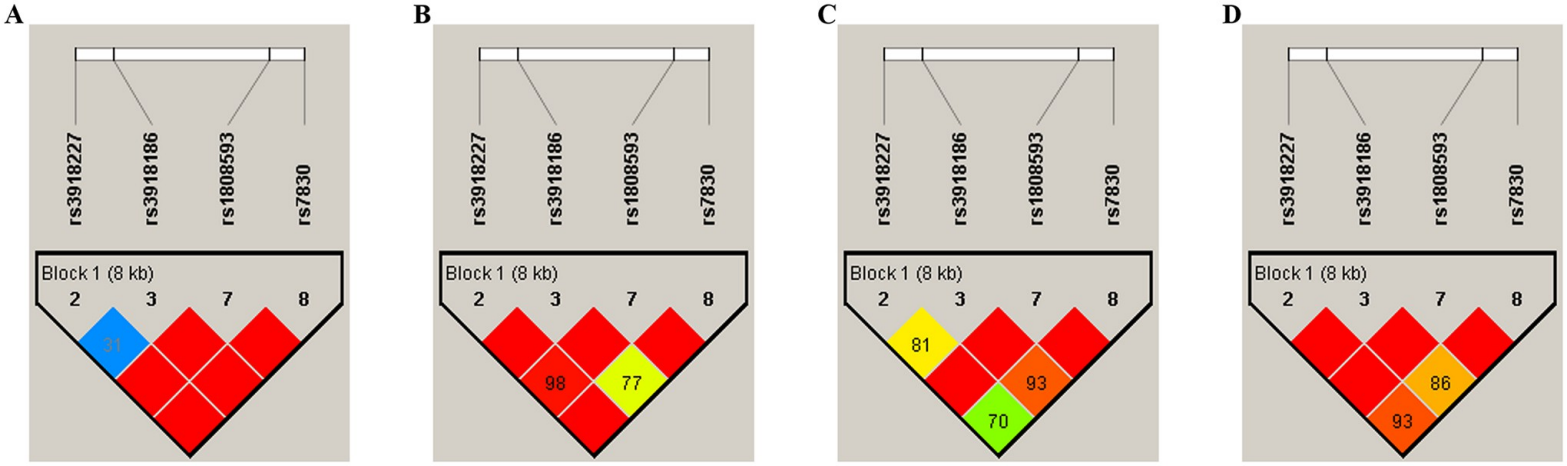
D’ values of the linkage disequilibrium pattern of the *NOS3* gene. (A) LD analysis of Guizhou Miao population. (B) LD analysis of Guizhou Buyi population. (C) LD analysis of Guizhou Han population. (D) LD analysis of Guizhou total populations.

LD analysis of SNPs was performed using Haploview 4.2. The results are shown in Fig 1; the D’ = 0 meant complete linkage equilibrium, D’ = 1 meant complete LD, and D’>0.8 meant strong LD represented by a red square. Strong LD was found for rs3918227, rs391818186, rs1808593 and rs7830. Haplotype analysis was performed using SHEsis online software. The results showed that compared with the non-haplotype CATT, the haplotype CATT was associated with an increased risk of hypertension both in the Guizhou Miao and Han populations (OR = 1.471, 95% CI: 1.010–2.143, *P* = 0.044 and OR = 1.692, 95% CI: 1.124–2.545, *P* = 0.011, respectively). Compared with non-haplotype CAGG, the risk of hypertension was reduced in the CAGG haplotype in the Guizhou Miao population (OR = 0.555, 95% CI: 0.330–0.934, *P* = 0.025), as shown in Table 5.

**Table 5:** Haplotype association analysis between EH group and control group in Guizhou population.

Nation	Haplotype ^a^	EH group (freq)	Control group (freq)	χ^2^	OR (95% CI) ^b^	*P* ^c^
Total populations	CATT	0.383	0.316	6.668	1.343 (1.073–1.687)	**0.010**
	CAGG	0.166	0.215	5.214	0.728 (0.554–0.957)	**0.022**
Miao population	CATT	0.505	0.410	4.061	1.471 (1.010–2.143)	**0.044**
	CAGG	0.123	0.201	5.010	0.555 (0.330–0.934)	**0.025**
Buyi population	CATT	0.284	0.286	0.003	0.988 (0.663–1.474)	0.954
	CAGG	0.175	0.183	0.051	0.947 (0.592–1.516)	0.821
Han population	CATT	0.367	0.255	6.418	1.692 (1.124–2.545)	**0.011**
	CAGG	0.197	0.262	2.588	0.694 (0.444–1.084)	0.108

^a^, Haplotype SNP combination rs3918227-rs391818186-rs1808593-rs7830;

^b^, Haplotype CATT compared to non-haplotype CATT. Other haplotypes are similar;

^c^, the *P* value of Chi-Square test.

### SNP-SNP interaction analysis

**Fig 2:**
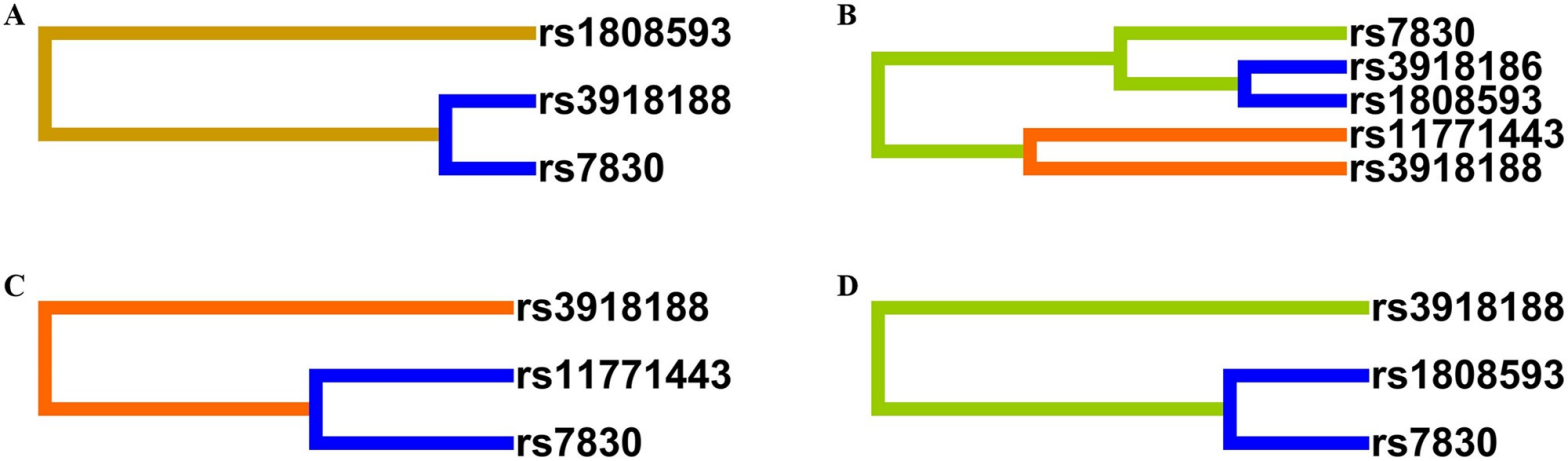
The dendograms of the SNP-SNP interaction in Guizhou populations. (A) SNP-SNP interaction of Miao population. (B) SNP-SNP interaction of Buyi population. (C) SNP-SNP interaction of Han population. (D) SNP-SNP interaction of Guizhou total populations.

The interaction effects of 8 SNPs in *NOS3* were analyzed using MDR software. The optimal interaction combination of SNPs was determined, including combination models of 1–3 SNPs, respectively. The best models for the three loci combinations for the total populations and the Miao population in Guizhou were rs3918188, rs1808593, and rs7830 (*P* < 0.001 and *P* < 0.001). The cross-validation consistency was both 7/10. The accuracy of training and testing balance were both more than 49%. The results suggested that rs3918188, rs1808593, and rs7830 had potential interaction with the occurrence of hypertension. The best models of the three loci combinations for the Buyi and Han populations in Guizhou were rs11771443, rs3918188, and rs7830 (*P* = 0.013 and *P* < 0.001). The cross-validation consistency was 4/10 and 5/10, respectively. The training and testing balance accuracy were both over 40%, implying that rs11771443, rs3918188, and rs7830 had a potential interaction with the occurrence of hypertension in the Guizhou Buyi and Han populations. The best models of loci 1 and 2 in different populations are also shown in Table 6. The dendograms of the SNPs interaction in the Guizhou population are shown in Fig 2.

**Table 6:** MDR analysis for SNP-SNP interaction in *NOS3* with EH risk.

Nation	Locus number	Model	Training Bal. Acc.	Testing Bal. Acc	CVC	χ^2^	*P* ^a^
Total populations	1	rs1808593	0.5500	0.5454	10/10	7.392	**0.007**
	2	rs1808593, rs7830	0.5615	0.5220	7/10	9.897	**0.002**
	3	rs3918188, rs1808593, rs7830	0.5751	0.5005	7/10	13.528	**<0.001**
Miao population	1	rs1808593	0.5846	0.5756	10/10	7.488	**0.006**
	2	rs1808593, rs7830	0.6079	0.5571	8/10	10.519	**0.001**
	3	rs3918188, rs1808593, rs7830	0.6337	0.4936	7/10	18.414	**<0.001**
Buyi population	1	rs1808593	0.5407	0.4521	6/10	1.450	0.229
	2	rs3918186, rs1808593	0.5655	0.4237	4/10	2.849	0.091
	3	rs11771443, rs3918188, rs7830	0.5897	0.4024	4/10	6.119	**0.013**
Han population	1	rs7830	0.5639	0.5194	9/10	6.213	**0.013**
	2	rs3918188, rs7830	0.6044	0.5222	9/10	8.756	**0.003**
	3	rs11771443, rs3918188, rs7830	0.6240	0.4570	5/10	12.343	**<0.001**

Abbreviations: Training Bal. Acc., Training Balanced accuracy; Testing Bal. Acc, Testing Balanced accuracy; CVC, Cross-Validation consistency. The training/testing balance accuracy represented the accuracy of the training set and the test set, and was used to evaluate the prediction error of the interaction model. Cross-validation consistency represented ten-fold cross-validation, comparing the number of times the same factor combination was determined, N/10 meant that N out of 10 cross-validations were significant.

^a^, *P* value of permutation test, *P* < 0.05 means that the model is statistically significant.

### Allele frequency difference

The allele frequencies of SNPs in Miao, Buyi and Han control groups in Guizhou were compared. At the same time, comparisons were made with the allele frequencies of Southern Han in the Ensemble database (https://asia.ensembl.org/index.html). The results showed that the allele frequencies of rs3918227 in Guizhou Miao were significantly different from the southern Han population (*P* < 0.05), while the allele frequencies of other SNPs were similar to the southern Han population. The allele frequencies of rs11771443 and rs7830 in Guizhou Miao were significantly different from Guizhou Buyi and Han populations (*P* < 0.05), especially rs7830 (*P* < 0.001), while there was no statistical difference in allele frequencies between Buyi and Han populations (Table 7). It indicated that the genetic characteristics of the Guizhou Miao population were relatively conservative, which may be caused by the relative lack of gene exchange with other ethnic groups during the migration process.

**Table 7:** Comparison of allele frequencies between Guizhou populations and Southern Han population.

SNP	Allele	Southern Han ^a^	Miao control	*P* ^b^	Buyi control	*P* ^b^	Han control	*P* ^b^	*P* ^c^	*P* ^d^	*P* ^e^
rs11771443	C	130(62%)	153(69%)	0.130	140(60%)	0.697	120(56%)	0.237	**0.043**	**0.006**	0.444
	T	80(38%)	69(31%)		94(40%)		94(44%)				
rs3918227	C	190(90%)	213(96%)	**0.033**	212(91%)	0.965	203(95%)	0.095	**0.026**	0.651	0.103
	A	20(10%)	9(4%)		22(9%)		11(5%)				
rs3918186	A	184(88%)	205(92%)	0.110	212(91%)	0.359	199(93%)	0.071	0.616	0.855	0.394
	T	26(12%)	17(8%)		22(9%)		15(7%)				
rs3918188	C	146(70%)	139(63%)	0.155	155(66%)	0.478	136(64%)	0.217	0.435	0.843	0.554
	A	64(30%)	83(37%)		79(34%)		78(36%)				
rs753482	C	9(4%)	3(1%)	0.081	3(1%)	0.076	4(2%)	0.169	0.948	0.720	0.714
	A	201(96%)	219(99%)		231(99%)		210(98%)				
rs891512	A	9(4%)	6(3%)	0.436	17(7%)	0.226	10(5%)	0.847	**0.032**	0.316	0.321
	G	201(96%)	216(97%)		217(93%)		204(95%)				
rs1808593	G	51(24%)	47(21%)	0.491	44(19%)	0.166	56(26%)	0.737	0.559	0.259	0.069
	T	159(76%)	175(79%)		190(81%)		158(74%)				
rs7830	G	121(58%)	108(49%)	0.067	149(64%)	0.206	143(67%)	0.057	**0.001**	**<0.001**	0.489
	T	89(42%)	114(51%)		85(36%)		71(33%)				

^a^, Data from Ensemble database;

^b^, Comparison between Miao, Buyi, Guizhou Han and southern Han respectively;

^c^, Comparison between Miao and Buyi;

^d^, Comparison between Miao and Guizhou Han;

^e^, Comparison between Buyi and Guizhou Han.

## Discussion

Guizhou is located in southwest China and has a monsoon climate, heavier rainfall and greater humidity than other Chinese provinces. It is a province with a diverse ethnic population, with the Miao and Buyi groups having the greatest non-Han populations. The unique geographical environment accounts for the local Guizhou population’s dietary preference for sour, spicy and oily food, smoking, and wine consumption. Hypertension is a multifactorial disease influenced by both genetics and the environment. Therefore, further research is warranted to study EH in the diverse ethnic groups in Guizhou.

The effect of *NOS3* gene polymorphism on hypertension and cardiovascular disease susceptibility has been extensively studied over the years [14–16]. In the present study, we substantiated the link between rs1808593 and rs7830 polymorphisms and EH in Guizhou populations. After stratification, the rs1808593 and rs7830 polymorphisms were linked to EH in the Guizhou Miao and Han populations, respectively. However, the rs11771443, rs3918227, rs3918186, rs3918188, rs753482, and rs891512 are not linked to EH. The haplotype of rs3918227-rs391818186-rs1808593-rs7830 is related to EH, and the rs3918188, rs1808593, and rs7830 polymorphisms have an interaction effect on EH.

Current evidence suggests that SNP rs1808593 is related to genetic susceptibility to hypertension in the Jiangsu population of China [17], consistent with the results of this study. In hypertensive patients, the rs891512, rs1808593, and rs1808593-rs7830 haplotypes were associated with the ankle-brachial index (a non-invasive indicator for peripheral artery disease) [18]. Metzger IF et al. [19] reported that the *NOS3* tag SNP rs3918188-rs743506-rs7830 haplotype is related to lower NO production and might be exploited as a cardiovascular risk marker. Interestingly, the present study showed that given the recessive inheritance pattern of rs7830 in the Han population, the risk of hypertension in males was lower than in females (OR = 0.537, 95%CI: 0.299–0.965, *P* = 0.038). Interestingly, a previous study showed that rs7830 is substantially associated with hypertension in Han women in Yunnan Province [20], which is in accordance with the results of this study. However, it has been reported that rs7830 is not associated with coronary artery disease (CAD) susceptibility [21]. De Miranda JA et al. [22] and Ingelsson E et al. [23] consistently found that *NOS3* label SNPs rs3918188 and rs7830 are not associated with hypertension in children and adolescents, and that rs3918186, rs3918188, rs753482, rs891512, rs1808593, rs7830 are not associated with endothelium-dependent vasodilation, which is similar to our results to a certain extent. It has long been thought that people with the rs753482-C genotype produced a novel stable truncated form of eNOS with altered enzyme activity, impacting NO generation and cellular function. These findings provide novel insights into various disorders involving NO responses in blood vessels [24]. Importantly, the present study provides hitherto undocumented evidence of the lack of correlation between rs753482 and EH.

The rs11771443 polymorphism has been associated with diabetic nephropathy in the Chinese Han population, and its genetic variation could be employed as a molecular marker to determine the risk of diabetic nephropathy [25]. Moreover, systolic blood pressure is positively correlated with chronic kidney disease and diabetes [26]. To the best of our knowledge, this is the first study to investigate the relationship between rs11771443 and EH. In contrast to the findings of this investigation, a meta-analysis revealed that rs891512 and rs11771443 gene polymorphisms might be employed as genetic indicators for CAD [27].

In European teenagers, the rs3918227 polymorphism has been linked to systolic blood pressure, and sufficient physical activity (≥60min/day) can reportedly mitigate the detrimental effects of the rs3918227 polymorphism on systolic blood pressure [28]. However, Grøntved A et al. [29] reported contrasting findings in their study, claiming that rs3918227 is not linked to blood pressure in European adolescents, which is in line with the findings of this study. Accordingly, the same SNP may yield different results in various ethnic populations, implying that *NOS3* may exhibit ethnic specificity in increasing susceptibility to EH.

Epidemiological studies suggest that 65%-78% of the risk of EH can be attributed to obesity [30]. Obesity, especially visceral obesity, is a major risk factor for hypertension and heart failure [31, 32]. Overweight and obesity were identified as risk factors for EH in the Guizhou population, consistent with the literature.

Hypertension is closely related to Alzheimer’s disease (AD), but the pathogenic mechanism between them is largely unclear [33]. NOS3-mediated oxidative stress is associated with amyloid-beta deposition, affecting AD development [34]. It has been reported that the NOS3 gene polymorphism rs1799983 may be associated with late-onset AD [35], while the association studies of rs1808593 and rs7830 with AD have not been reported. Therefore, this study provides genetic data evidence based on population surveys and found that rs1808593 and rs7830 may be associated with EH, which not only provides an important genetic reference value for the genetic mechanism, clinical prevention, and treatment of hypertension but also provides new ideas for the genetic association study between AD and EH. Future studies should apply the Chromatin immunoprecipitation (ChIP) assay to detect the binding activity of these genotypes or the function of these polymorphisms with a luciferase reporter assay.

Several limitations and shortcomings were present in this study. Given the retrospective nature of this study, blood parameters, including fasting blood glucose, total cholesterol, triacylglycerol, low-density lipoprotein, and other biochemical indicators closely related to hypertension, were unavailable for the Miao and Buyi populations. Hence, the present study could not analyze their potential association with hypertension. It is widely acknowledged that EH is a complex chronic disease involving the interaction between genes and the environment. The living environment of different ethnic groups (such as living standards and geographical environment) may have an important impact on the relationship between susceptibility genes and EH. Moreover, other factors such as smoking, dietary habits, and educational level were unavailable in the present study, and their potential interaction with genes could not be ascertained. In addition, regression analysis showed no significant association between gender and EH, which may be due to the gender error in sample collection caused by men’s annual outing to work. Therefore, the relationship between *NOS3* gene polymorphisms and EH warrants further investigation in a larger and more comprehensive population.

Taken together, this is the first study to use the Sequenom MassARRAY platform to investigate the association between eight SNPs of the *NOS3* gene and EH in the Guizhou populations. Importantly, we found that age, sex, and obesity are risk factors for EH in the Guizhou ethnic populations. SNPs rs1808593 and rs7830 are linked to EH in the Guizhou populations, exhibiting ethnic differences.

## Supporting information

S1 Table Primer sequences for *NOS3* gene SNP genotyping. (DOCX)

S2 Table Biochemical indices of Guizhou Han population (n = 221). (DOCX)

S3 Table FDR corrected *P* values for alleles and genotypes. (DOCX)
