# Supplementary material for: Polymorphism of NOS3 gene and its association with essential hypertension in Guizhou populations of China
Source: PLoS One. 2023 Feb 9;18(2):e0278680. doi: 10.1371/journal.pone.0278680 (PMC9910734; doi:10.1371/journal.pone.0278680)
Supplement: S1 Table — (DOCX) [file pone.0278680.s001.docx]

| **S1 Table Primer sequences for *NOS3* gene SNP genotyping** | |
| --- | --- |
| **SNP** | **Primer sequence (5' to 3')** |
| rs11771443 | Upstream primer: ACGTTGGATGGAAGGATCAGGCCCACAATG |
|  | Downstream primer: ACGTTGGATGCGTCTGTGGGCGTAACATC |
|  | Single base extension primer: ggacCATCCCCCGCTGGGCTGATGT |
| rs3918227 | Upstream primer: ACGTTGGATGTTCATAATAGCCCCGACCTG |
|  | Downstream primer: ACGTTGGATGTGAGTGCCGTTCATTGTGTG |
|  | Single base extension primer: GTGAATATCCCCAGTTTGTTTAC |
| rs3918186 | Upstream primer: ACGTTGGATGGGAAGGAAGCTGGAAGGAAC |
|  | Downstream primer: ACGTTGGATGTCGTTCTTGCTAACTCTGGC |
|  | Single base extension primer: cTCTGCAGTTGAGCACC |
| rs3918188 | Upstream primer: ACGTTGGATGACTTCACTGAGACTGAAGGG |
|  | Downstream primer: ACGTTGGATGAAAAGTGGGAGCAAGGCACA |
|  | Single base extension primer: agcacGGCACACGTACAAGGG |
| rs753482 | Upstream primer: ACGTTGGATGAGAATGTGGGAGGAGGAGG |
|  | Downstream primer: ACGTTGGATGAACCTGCAAAGCAGCAAGTC |
|  | Single base extension primer: aggagGGACGACGGCTTTACCGCCCCCC |
| rs891512 | Upstream primer: ACGTTGGATGACCCTGTGCCCTGCTTCATC |
|  | Downstream primer: ACGTTGGATGAGTGACACTCCTCACCAAAG |
|  | Single base extension primer: AGCTCCCCACCAAGTC |
| rs1808593 | Upstream primer: ACGTTGGATGAAACTATAGCTCCCAGAGCC |
|  | Downstream primer: ACGTTGGATGGGACATAGAGAGGAACACAG |
|  | Single base extension primer: gacaaAGGGCCAGCCGGTTTGAT |
| rs7830 | Upstream primer: ACGTTGGATGAGCGGCTGCATGACATTGAG |
|  | Downstream primer: ACGTTGGATGGTCCCTAGATTGTGTGACTC |
|  | Single base extension primer: agacaTTCAGGCAGTCCTTTAGTC |
