# Supplementary material for: Polymorphism of NOS3 gene and its association with essential hypertension in Guizhou populations of China
Source: PLoS One. 2023 Feb 9;18(2):e0278680. doi: 10.1371/journal.pone.0278680 (PMC9910734; doi:10.1371/journal.pone.0278680)
Supplement: S2 Table — (DOCX) [file pone.0278680.s002.docx]

| **S2 Table Biochemical indices of Guizhou Han population (n=221)** | | | | |
| --- | --- | --- | --- | --- |
| **Clinical index** | **Control (n=107)** | **EH (n=114)** | **t** | ***P*** |
| Fasting blood-glucose (mmol/L) | 5.02±0.72 | 5.94±1.93 | -4.68 | **<0.001** |
| Cholesterol (mmol/L) | 5.29±0.78 | 5.54±1.04 | -2.03 | **0.044** |
| Triglyceride (mmol/L) | 1.98±1.12 | 2.70±1.51 | -4.03 | **<0.001** |
| Measurement data were expressed as mean±SD, and Student’s t-test was used for comparison between groups. | | | | |
