# Supplementary material for: Polymorphism of NOS3 gene and its association with essential hypertension in Guizhou populations of China
Source: PLoS One. 2023 Feb 9;18(2):e0278680. doi: 10.1371/journal.pone.0278680 (PMC9910734; doi:10.1371/journal.pone.0278680)
Supplement: S3 Table — (DOCX) [file pone.0278680.s003.docx]

| **S3 Table FDR corrected *P* values for alleles and genotypes.** | | | | | | | | | | | | | | | | |
| --- | --- | --- | --- | --- | --- | --- | --- | --- | --- | --- | --- | --- | --- | --- | --- | --- |
| **SNP** | **Total populations** | | | | **Miao population** | | | | **Buyi population** | | | | **Han population** | | | |
|  | ***P1*** | ***P1*.adjust** | ***P2*** | ***P2*.adjust** | ***P1*** | ***P1*.adjust** | ***P2*** | ***P2*.adjust** | ***P1*** | ***P1*.adjust** | ***P2*** | ***P2*.adjust** | ***P1*** | ***P1*.adjust** | ***P2*** | ***P2*.adjust** |
| rs1808593 | **0.013** | 0.064 | **0.023** | 0.14 | **0.015** | 0.12 | **0.022** | 0.176 | 0.812 | 0.959 | 0.230 | 0.776 | 0.114 | 0.456 | 0.228 | 0.716 |
| rs7830 | **0.016** | 0.064 | **0.035** | 0.14 | 0.071 | 0.284 | 0.192 | 0.747 | 0.924 | 0.959 | 0.950 | 1 | **0.019** | 0.152 | **0.038** | 0.304 |
| rs891512 | 0.283 | 0.69 | 0.366 | 0.586 | 0.285 | 0.57 | 0.280 | 0.747 | 0.959 | 0.959 | 1.000 | 1 | 0.312 | 0.624 | 0.495 | 0.716 |
| rs3918227 | 0.345 | 0.69 | 0.218 | 0.506 | 0.800 | 0.977 | 0.796 | 0.909 | 0.511 | 0.959 | 0.291 | 0.776 | 0.649 | 0.722 | 0.537 | 0.716 |
| rs3918188 | 0.569 | 0.91 | 0.696 | 0.928 | 0.194 | 0.517 | 0.415 | 0.83 | 0.338 | 0.959 | 0.553 | 1 | 0.484 | 0.722 | 0.511 | 0.716 |
| rs11771443 | 0.695 | 0.927 | 0.856 | 0.957 | 0.419 | 0.67 | 0.715 | 0.909 | 0.778 | 0.959 | 0.896 | 1 | 0.246 | 0.624 | 0.310 | 0.716 |
| rs3918186 | 0.921 | 0.958 | 0.253 | 0.506 | 0.855 | 0.977 | 0.556 | 0.889 | 0.923 | 0.959 | 0.166 | 0.776 | 0.722 | 0.722 | 0.781 | 0.781 |
| rs753482 | 0.958 | 0.958 | 0.957 | 0.957 | 1.000 | 1 | 1.000 | 1 | 0.724 | 0.959 | 0.722 | 1 | 0.717 | 0.722 | 0.715 | 0.781 |
| Abbreviations: FDR, false discovery rate. *P1* is the allele *P* value, and *P2* is the genotype *P* value.  R code: data <- c (0.038,0.228,0.31,0.495,0.511,0.537,0.715,0.781) p. adjust (data, method = 'fdr', n = length(data)) | | | | | | | | | | | | | | | | |
